# Dr. Curry, on Sudden Death

**Published:** 1816-03

**Authors:** James Curry

**Affiliations:** Bridge Street, Blackfriars


*To the Editors of the Medico-Chirurgical Journal & Review.*

GENTLEMEN,

I AM happy to announce, that the conjecture I had thrown out, respecting the cause of sudden death in many cases, (*Observations on Apparent Death, Appendix, p.* 153) has lately received an additional testimony in its favour, which some persons may consider as entitled to more attention than any thing coming from me. Dr. Odier, of Geneva, who translated the first edition, has lately done the same with all the new matter contained in the second, published in 1815. In a letter which I have recently received from him, he says—"You may see at the end (of the printed extracts sent by him) a Note, in which I have related a case of sudden death, which confirms *your* opinion on the principal cause of it. I have seen many other cases, in which, on opening the *head*, I found nothing preternatural; but not suspecting the *stomach* or *intestines*, I did not minutely inquire whether they were the seat of the disease: they would, probably, have afforded farther proofs."

The following is the Note alluded to, in Dr. Odier's own words:

" Je fus appelé un jour à 7 h. du matin, au secours d'une dame, agée de 72 ans, que j'avois vue la veille parfaitement bien portante, et qui venoit de se trouver mal. J'arrivai au moment où elle expiroit. On me raconta q'une demi-heure auparavant, apres avoir passée une tres-bonne nuit, elle s'etoit plainte a son reveil d'une vive douleur a l'estomac, avec quelques nausées. Elle avoit demandé une infusion de melisse, et mourut en la buvant. J'avois ouï dire a Londres, au Dr. George Fordyce, qu'il avoit vu des cas de mort subite, occasionnés par l'irritation que cause quelquefois le passage d'un calcul biliare au travers du conduit choledoque. La douleur dont s'etoit plainte la malade un instant avant sa mort, me fit soupçonner ici quelque-chose de semblable. Je demandai, et j'obtins l'ouverture du cadavre. Nous trouvames tout le corps en tres bon etat, a l'exception du conduit choledoque, dont l'interieur nous parut fort inflammé, et portoit evidemment les marques d'une grande irritation. Cependant nous ne decouvrimes ni dans l'intestin, ni dans aucune partie du canal alimentaire, aucun vestige de calcul. Mais il y en avoit quelques-uns dans la vesicule du fiel; et il ne me paroit pas impossible, que l'un d'eux y eut reflué, apres s'etre engagé dans le conduit."

The case just related, although it will be generally allowed to refer the source of irritation to the same organs and parts that I had pointed out, yet has some obscurity attached to it, from there being no particular notice taken of the matters discovered in the duodenum, and which I have stated to be *dark green viscid bile*. That a gall-stone so small as to escape detection, should, in its passage thro' the ducts into the intestine, have produced such *mechanical* irritation as to occasion *sudden* death, is not countenanced by any analogous fact within my recollection; and still less, that an irritation adequate to that effect, should be produced by a gall-stone dropping into the cystic duct, and then falling back again into the gall-bladder. The learned and ingenious narrator does not say whether this case occurred before or after his becoming acquainted with my opinion; and it is obvious, that if before that event, he might not pay any attention to the colour and other qualities of the matter discovered in the duodenum, but have rested satisfied upon not finding any biliary calculus that had been expelled.

I trust, however, that this additional testimony bearing on the general question, will soon bring forth something more pointed, either to confirm or refute my conjecture.

---

The gentleman who reviewed my Observations on Apparent Death, expresses his despair of my redeeming the pledge which I gave several years ago, to publish a work upon hepatic diseases. I certainly regret, and more than any one else can, that a variety of causes have contributed to delay this publication; but at the same time can assure the Reviewer, that the intention has never been abandoned, and that I am now occupied upon the Work (which, from being merely observations on disorders obviously referable to the liver, has expanded itself into a general pathology) as much as my avocations as a teacher and practitioner will possibly admit. It may not, perhaps, be known to him, and to many others, who feel the same impression, that I suffered not less than two years of distressing illness, and a corresponding duration of incapacity for every thing which required either bodily or mental exertion, from having taken, though in very moderate quantities, the Eau medicinale; and that my personal experience, together with the result of my inquiries on the effects of that article, will probably be the first fruits of my recovered health.

I am, &c.

JAMES CURRY, M. D.

Bridge Street, Blackfriars,
Jan. 12, 1816.

